# Zika Virus Infection of Human Iris Pigment Epithelial Cells

**DOI:** 10.3389/fimmu.2021.644153

**Published:** 2021-04-22

**Authors:** Feargal J. Ryan, Jillian M. Carr, João M. Furtado, Yuefang Ma, Liam M. Ashander, Milena Simões, Genevieve F. Oliver, G. Bracho Granado, Abby C. Dawson, Michael Z. Michael, Binoy Appukuttan, David J. Lynn, Justine R. Smith

**Affiliations:** ^1^ Precision Medicine Theme, South Australian Health & Medical Research Institute, Adelaide, SA, Australia; ^2^ Flinders University College of Medicine and Public Health, Bedford Park, SA, Australia; ^3^ Ophthalmology Division, Ribeirão Preto Medical School, University of São Paulo, Ribeirão Preto, Brazil

**Keywords:** Zika, virus, ZIKV, human, eye, iris, epithelium, pigment epithelial cell

## Abstract

During recent Zika epidemics, adults infected with Zika virus (ZIKV) have developed organ-specific inflammatory complications. The most serious Zika-associated inflammatory eye disease is uveitis, which is commonly anterior in type, affecting both eyes and responding to corticosteroid eye drops. Mechanisms of Zika-associated anterior uveitis are unknown, but ZIKV has been identified in the aqueous humor of affected individuals. The iris pigment epithelium is a target cell population in viral anterior uveitis, and it acts to maintain immune privilege within the anterior eye. Interactions between ZIKV and human iris pigment epithelial cells were investigated with infectivity assays and RNA-sequencing. Primary cell isolates were prepared from eyes of 20 cadaveric donors, and infected for 24 hours with PRVABC59 strain ZIKV or incubated uninfected as control. Cytoimmunofluorescence, RT-qPCR on total cellular RNA, and focus-forming assays of culture supernatant showed cell isolates were permissive to infection, and supported replication and release of infectious ZIKV. To explore molecular responses of cell isolates to ZIKV infection at the whole transcriptome level, RNA was sequenced on the Illumina NextSeq 500 platform, and results were aligned to the human GRCh38 genome. Multidimensional scaling showed clear separation between transcriptomes of infected and uninfected cell isolates. Differential expression analysis indicated a vigorous molecular response of the cell to ZIKV: 7,935 genes were differentially expressed between ZIKV-infected and uninfected cells (FDR < 0.05), and 99% of 613 genes that changed at least two-fold were up-regulated. Reactome and KEGG pathway and Gene Ontology enrichment analyses indicated strong activation of viral recognition and defense, in addition to biosynthesis processes. A CHAT network included 6275 molecular nodes and 24 contextual hubs in the cell response to ZIKV infection. Receptor-interacting serine/threonine kinase 1 (RIPK1) was the most significantly connected contextual hub. Correlation of gene expression with read counts assigned to the ZIKV genome identified a negative correlation between interferon signaling and viral load across isolates. This work represents the first investigation of mechanisms of Zika-associated anterior uveitis using an *in vitro* human cell model. The results suggest the iris pigment epithelium mounts a molecular response that limits intraocular pathology in most individuals.

## Introduction

Zika virus (ZIKV) is a single-stranded RNA flavivirus, which was first isolated from non-human primates in Uganda in 1947, and from humans in the same country in the early 1950s (1). Zika virus disease is generally considered to pose little risk to human health unless it is contracted *in utero*. In recent epidemics from 2013 to 2014 in French Polynesia, and from 2015 to 2016 in Brazil and other American nations, women who were infected while pregnant delivered infants with a spectrum of developmental abnormalities (2, 3), now referred to as congenital Zika syndrome (4). Characteristic abnormalities in this disease include severe microcephaly, thinned cerebral cortex with subcortical calcification, extrapyramidal motor disturbances and contractures. In addition, approximately 70% of babies with congenital Zika syndrome develop ocular abnormalities, including retinal dystrophic lesions that show a preference for the macula, and therefore cause blindness (5). However, in some adults, infection with ZIKV results in serious inflammatory conditions (1).

Uveitis –or intraocular inflammation– represents a diverse spectrum of diseases, classified according to anatomical localization within the eye and by specific phenotype (6). Different types of uveitis have been reported in adult patients infected with ZIKV (7). The most common form is anterior uveitis –based in the iris and ciliary body– which occurs in approximately 50% of patients who present with “red eyes” in the context of Zika fever (8). This uveitis is typically bilateral, may impact the visual acuity, and often is accompanied by an elevation of intraocular pressure, as we (9) and others (8, 10, 11) have documented (**Table 1**). In one of the first reports of Zika-associated anterior uveitis, aqueous humor was collected from the eye of a patient treated with corticosteroid eye drops alone; ZIKV RNA was recovered from fluid taken at 8 days after the onset of systemic symptoms, but not at 16 days (9). This implies that Zika-associated anterior uveitis is associated with the presence of ZIKV inside the eye.

**Table 1:** Clinical features of ZIKV-associated anterior uveitis diagnosed in patients presenting to the Ophthalmology Clinic of Ribeirão Preto General Hospital, Ribeirão Preto, São Paulo or reported in the medical literature.

Location; Demographics (Reference if applicable)*	Clinical features	Basis for diagnosis	Treatment and course
Ribeirão Preto, Brazil; M, 40 years (9)	OD/OS; VA=20/20 OD, 20/60 OS; AC cell=0.5+ OD, 2+ OS; IOP=16mmHg OD, 15mmHg OS; other=punctate keratitis	Epidemiology; Systemic features; Serology; Aqueous and blood RT-PCR	Topical glucocorticoid; Remission of uveitis with improvement of VA
Ribeirão Preto, Brazil; F, 55 years	OD/OS; VA=20/30 OD, 20/25 OS; AC cell=3+ OD, 2+ OS; IOP=34mmHg OD, 17mmHg OS	Epidemiology; Systemic features; Aqueous and blood RT-PCR	Topical glucocorticoid and hypotensive; Remission of uveitis with improvement of VA
Ribeirão Preto, Brazil; F, 41 years	OD; VA=20/100 OD, 20/20 OS (unrelated corneal dystrophy OD); AC cell=3+ OD, 0+ OS; IOP=17mmHg OD, 16mmHg OS	Epidemiology; Systemic features; Negative work-up for other causes	Topical glucocorticoid; Remission of uveitis with no change in VA
Ribeirão Preto, Brazil; F, 40 years	OS; VA=20/100 OD, 20/30 OS (unrelated toxoplasmic retinal scar OD); AC cell=0+ OD, 1+ OS; IOP=16mmHg OD, 16mmHg OS	Epidemiology; Systemic features; Negative work-up for other causes	Topical glucocorticoid; Remission of uveitis with improvement of VA
Ribeirão Preto, Brazil; F, 66 years	OD; VA=20/40 OD, 20/30 OS; (unrelated macular hole OD); AC cell=2+ OD, 0+ OS; IOP=14mmHg OD, 14mmHg OS	Epidemiology; Systemic features; Negative work-up for other causes	Topical glucocorticoid; Remission of uveitis with no change in VA
Rio de Janeiro, Brazil; M, 39 years (10)	OD/OS; VA=20/40 OD, 20/40 OS; AC cell=1+ OD, 1+ OS; IOP=40mmHg OD, 28mmHg OS	Epidemiology; Systemic features; Negative work-up for other causes	Topical glucocorticoid and hypotensive; Remission of uveitis with improvement of VA and lowering of IOP
Martinique, France; W, 50s (11)	OD/OS; VA=20/25 OD, 20/20 OS; AC cell present (grade NS); IOP=36mmHg OD, 26mmHg OS	Epidemiology; Systemic features; Urine RT-PCR; Negative work-up for other causes	Topical hypotensive; Remission of uveitis and lowering of IOP#
Martinique, France; W, 60s (11)	OD/OS; VA=20/40 OD, 20/20 OS; AC cell present (grade NS); IOP=58mmHg OD, 32mmHg OS; other=corneal edema	Epidemiology; Systemic features; Aqueous and urine RT-PCR; Negative work-up for other causes	Topical hypotensive; Remission of uveitis and lowering of IOP
Guadeloupe, France; F, adult (age NS) (8)	OD/OS; VA=20/20 OD, 20/25 OS; AC cell=0.5+ OD, 1+ OS; IOP normal (value NS)	Epidemiology; Systemic features; Aqueous RT-PCR; Negative work-up for other causes	Topical glucocorticoid; Remission of uveitis and recovery of VA
Guadeloupe, France; M, adult (age NS) (8)	OD/OS; VA=20/20 OD, 20/25 OS; AC cell=0 OD, 0.5+ OS; IOP=16mmHg OD, 28mmHg OS	Epidemiology; Systemic features; Aqueous RT-PCR; Negative work-up for other causes	Topical glucocorticoid and hypotensive; Remission of uveitis; Persistent elevation of IOP
Guadeloupe, France; Gender NS, adult (Age NS) (8)	OD/OS; VA=20/32 OD, 20/20 OS; AC cell=0 OD, 1+ OS; IOP=25mmHg OD, 16mmHg OS	Epidemiology; Systemic features; Aqueous RT-PCR; Negative work-up for other causes	Topical glucocorticoid and hypotensive; Remission of uveitis; Persistent elevation of IOP
Guadeloupe, France; Gender NS, adult (Age NS) (8)	OD/OS; VA=20/20 OD, 20/40 OS; AC cell=0 OD, 1+ OS; IOP=16mmHg OD, 30mmHg OS	Epidemiology; Systemic features; Aqueous RT-PCR; Negative work-up for other causes	Topical glucocorticoid and hypotensive; Remission of uveitis; Persistent elevation of IOP

AC, anterior chamber; F, female; IOP, intraocular pressure; M, male; NS, not specified; OD, right eye; OS, left eye; RT-PCR, reverse transcription-polymerase reaction; VA, visual acuity.

*Reference 8 reports an observational study of 62 patients that included case histories from 4 patients whose diagnoses were based on aqueous RT-PCR plus other tests for Zika. Results of other tests were not specified. Visual acuity outcome was not stated for 3 of the 4 cases.

Several independently conducted studies have explored the pathogenic mechanisms of congenital Zika syndrome in mouse models (12–15), and human retinal cells or cell lines (16–19). However, the pathogenic mechanisms that are responsible for Zika-associated anterior uveitis have not been investigated previously. In the anterior eye, iris pigment epithelial cells play a major role in controlling the immune microenvironment (20). In herpes viral anterior uveitis, which is the most studied viral anterior uveitis, pathological changes involving these cells result in a variety of gross ocular changes, including tissue defects that may impact iris appearance (21). To address the important knowledge gap around the pathogenesis of Zika-associated anterior uveitis, we investigated interactions between ZIKV and primary human iris pigment epithelial cells using infectivity assays and next generation sequencing of cellular RNA content (RNA-Seq).

## Methods

### Human Iris Pigment Epithelial Cells

Iris pigment epithelial cell isolates were prepared from paired human cadaver donor eyes, using methods that were adapted from the technique published by Mai et al. (22). Irises were carefully dissected from the two posterior eyecups, digested in 0.25% trypsin (Thermo Fisher Scientific-Gibco, Grand Island, NY) for 45 minutes at 37°C, and transferred to phosphate buffered saline (PBS) with 2% fetal bovine serum (FBS; Bovogen Biologicals, Keilor East, Australia) at room temperature. Pigment epithelial cells were gently brushed from the tissue using the bent tip of a Pasteur pipette. The cell suspension was pelleted at 10 x g for 5 minutes at room temperature, washed with PBS with 2% FBS, and re-suspended in Epithelial Cell Medium (ScienCell Research Laboratories, Carlsbad, CA; catalog number 4101, consisting of basal medium, 2% FBS, Epithelial Cell Growth Supplement and a penicillin-streptomycin solution). Cells were expanded at 37°C and 5% CO_2_ in air, with medium refreshed twice a week, initially in a 6 cm diameter dish, subsequently trypsin-passaged and transferred to a T75 flask, and finally stored frozen in liquid nitrogen on reaching confluence. Cell phenotype was confirmed for all cell isolates by immunocytochemical detection of cytokeratin-8 indicating epithelial lineage and absence of α-smooth muscle actin, which is expressed during mesenchymal differentiation (see *Cytoimmunofluorescence*). All cell isolates were demonstrated to be free of *Mycoplasma* species contamination by quantitative real-time polymerase chain reaction (qPCR) of DNA extracted from culture supernatant.

### Zika Virus

Zika virus (strain PRVABC59: National Center for Biotechnology Information Nucleotide (GenBank) Accession Number, KU501215; originally isolated from human serum in Puerto Rico in 2015) was propagated in C6/36 mosquito cells (American Type Culture Collection [ATCC], Gaithersburg, MA) that were grown in Eagle’s basal medium, 1 mM sodium pyruvate, 1x MEM Non-Essential Amino Acids Solution, 2 mM L-glutamine and 10% FBS (all from Thermo Fisher Scientific-Gibco), and transferred to a medium modified by the removal of the amino acid supplement and reduction of FBS to 7.5%, and with the addition of 10 mM HEPES, ahead of infection. Virus stocks were titered by plaque assay on Vero cells, with plaques detected by neutral red overlay and expressed as plaque forming units/mL, as previously published (23).

### Viral Infection of Human Iris Pigment Epithelial Cells

Human iris pigment epithelial cells were rapidly thawed and plated in 10 cm diameter tissue culture dishes in Epithelial Cell Medium with 2% FBS. After cells reached confluence, they were subcultured to 6 cm dishes (2 x 10^6^ cells/dish) or 6-well plates, including wells with coverslips (2 x 10^5^ cells/well), and rested overnight. Subconfluent monolayers of iris epithelial cells were infected with ZIKV at multiplicity of infection (MOI) of 1 or 5, or mock-infected, in a minimum amount of FBS-free Dulbecco’s Modified Eagle Medium (DMEM, Thermo Fisher Scientific-Gibco; catalog number 11965). After 90 minutes, the inoculum was removed, and the infected cultures were returned to incubation in fresh Epithelial Cell Medium for a total of 24 hours, when cells were either lyzed with TRIzol Reagent (Thermo Fisher Scientific-Invitrogen, Carlsbad, CA) and stored at -80°C ahead of RNA extraction, or fixed with 2% paraformaldehyde for 30 minutes and stored in 70% ethanol at 4°C for cytoimmunofluorescence. Culture supernatant was snap-frozen at -80°C. A 24-hour time interval was selected to obtain a comprehensive picture of the host cell response to replicating virus. All work with live virus was conducted in a Physical Containment Level 2 Facility, under a Microbiological Dealing issued by the Flinders University Institutional Biosafety Committee (dealing number: 2016-07.1).

### Focus Forming Assay

Supernatants from ZIKV-infected and uninfected human iris pigment epithelial cell monolayers were tested for infectious virus with a high throughput plaque assay performed on an Operetta High-Content Analysis System (PerkinElmer, Waltham, MA), modifying a previously reported method (24). In brief, Vero cells were seeded at 1 x 10^4^ cell/well in 96-well plates and rested overnight, subsequently exposed in triplicate to serially diluted supernatant –or medium only control or ZIKV (3 x 10^6^ pfu/mL) positive control– for 90 minutes, and finally incubated in fresh medium for 24 hours. Cell monolayers were fixed with 2% paraformaldehyde, permeabilized with 0.05% IGEPAL CA-630, and labeled with mouse anti-flavivirus envelope protein (ENV) antibody (ATCC HB-112TM hybridoma [clone 4G2] culture supernatant diluted 1:10, applied overnight at 4 °C), followed by Alexa Fluor 488-tagged donkey anti-mouse immunoglobulin antibody (Thermo Fisher Scientific-Molecular Probes, Eugene, OR; catalog number A21202; working concentration, 10 µg/mL, applied for 1 hour at room temperature) with Hoechst 33342 (2’-[4-ethoxyphenyl]-5-[4-methyl-1-piperazinyl]-2,5’-bi-1H-benzimidazole trihydrochloride trihydrate) counterstain. Wells were imaged using the Operetta High-Content Analysis System (11 fields/well, 100x magnification), and images were analyzed using the Columbus Image Data Storage and Analysis v2.7 (PerkinElmer) to quantify the number of labeled infected cell clusters per well. Data was processed in Vortex v2014.03.71496.59-s (Dotmatics, Bishops Stortford, UK), including calculation of focus forming units/mL for each supernatant sample and dilution.

### Cytoimmunofluorescence

Human iris pigment epithelial cell monolayers were fixed with 2% or 4% paraformaldehyde in preparation for cytoimmunofluorescence labeling. For cell phenotyping, monolayers were labeled overnight at 4°C with one of following rabbit polyclonal antibodies diluted in 0.05% Triton X-100 and 2% bovine serum albumin in PBS: anti-human cytokeratin 8 (Abcam, Cambridge, United Kingdom; catalog number ab53280; working dilution, 1:250, equivalent to 0.132 µg/mL), α-smooth muscle actin (Abcam, catalog number ab5694; working dilution, 1:100, equivalent to 2 µg/mL) and rabbit immunoglobulin (Vector Laboratories, Burlingame, CA; catalog number I-1000, working concentration, 2 µg/mL). Cell monolayers were incubated with Alexa Fluor 488-tagged donkey anti-rabbit immunoglobulin antibody (Thermo Fisher Scientific-Molecular Probes; catalog number A11008; working concentration, 1 µg/mL) for 1 hour at room temperature, counterstained with DAPI (4′,6-diamidino-2-phenylindole) for 2 minutes, and imaged by fluorescence microscopy at 200x magnification. For demonstration of viral infection, ZIKV-infected and uninfected cell monolayers were labeled for 1 hour at room temperature with mouse anti-flavivirus envelope protein antibody, used as hybridoma culture supernatant diluted 1:10 in Hanks’ balanced salt solution with 2% goat serum. Cell monolayers were incubated with Alexa Fluor 488-tagged goat anti-mouse immunoglobulin antibody (Thermo Fisher Scientific-Molecular Probes; catalog number A11001; working concentration, 13 µg/mL) for 30 minutes at room temperature, counterstained with Hoechst 33342 for 10 minutes, and imaged by confocal microscopy at 100x magnification.

### RNA Extraction

Total RNA was recovered from ZIKV-infected and uninfected iris pigment epithelial cells by phenol/chloroform extraction using Trizol Reagent, according to the manufacturer’s protocol. RNase-free glycogen was added to all samples during precipitation to maximize RNA yields. RNA was frozen at -80°C prior to use for RNA-Seq and reverse transcription (RT)-qPCR. Nucleic acid concentrations were determined using the Qubit 2.0 Fluorometer (Thermo Fisher Scientific-Life Technologies, Carlsbad, CA).

### Reverse Transcription-Polymerase Chain Reaction

Reverse transcription was carried out using iScript Reverse Transcription Supermix for RT-qPCR (Bio-Rad, Hercules, CA). Total RNA input was 500 ng per reaction, yielding 20 μl of cDNA which was diluted 10-fold ahead of use in qPCR.

Quantitative real-time PCR was performed using the CFX Connect Real Time PCR Detection System (Bio-Rad), and a reaction mix consisting of 4 μL of iQ Sybr Green Supermix or SsoAdvanced SYBR Green Supermix (Bio-Rad), 1.5 μL each of 20 μM forward and reverse primer, 11 μL of nuclease-free water and 2 μL of diluted cDNA. Primer sequences (and expected product sizes) were as follows: ZIKV ENV forward 5’-GCTGGDGCRGACACHGGRACT-3’, reverse 5’-RTCYACYGCCATYTGGRCTG-3’ (304 bp); interferon-β (IFN-β) forward 5’-AAACTCATGAGCAGTCTGCA-3’, reverse 5’-AGGAGATCTTCAGTTTCGGAGG-3’ (168 bp); peptidylprolyl isomerase A (PPIA) forward 5’-GAGCACTGGAGAGAAAGGATTT-3’, reverse 5’-GGTGATCTTCTTGCTGGTCTT-3’ (355 bp); and ribosomal protein lateral stalk subunit P0 (RPLP0) forward 5’-GCAGCATCTACAACCCTGAA-3’, reverse 5’-GCAGATGGATCAGCCAAGAA-3’ (235 bp). Primer efficiency for all primer sets was greater than 85.0% except in the case of ZIKV ENV, for which efficiency was 76.5%. Melting curves were included in each RT-qPCR run to confirm a single peak was produced, and amplicon sizes for all primers were confirmed by agarose gel electrophoresis.

Absolute quantification of viral genome equivalents was carried out by generating a standard curve for ZIKV ENV using purified PCR product serially diluted 10-fold from an initial concentration of 10 pg/μL. Starting quantity in ng (SQ) was determined for ZIKV-infected and uninfected samples using CFX Manager software v3.1 (Bio-Rad). Viral genome equivalent number was calculated using the equation ([SQ/10^9^]/[bp molecular mass x product size]) x 6.02214 x 10^23^. Relative expression of IFN-β was determined by the Pfaffl method (25) and normalized to two reference genes –PPIA and RPLP0– which were stable as indicated by CV-value less than 0.25 and M-value less than 0.5.

### RNA Sequencing

An RNA integrity number of at least 8 was confirmed on the LabChip GX Touch 24 Nucleic Acid Analyzer (Perkin Elmer, Hopkinton, MA). The cDNA libraries were prepared with the TruSeq Stranded Total RNA Kit (Illumina, San Diego, CA), strictly in accordance with the manufacturer’s instructions. Pooled libraries were sequenced on the NextSeq 500, with the High-Output Kit for 2 x 75 cycles of sequencing, and aiming for an average depth of 50 x 10^6^ reads per sample. The PhiX Control v3 library (Illumina) was used as a sequencing control.

### Processing and Analysis of RNA Sequencing Data

Quality of the data obtained by RNA sequencing was assessed with FastQC v0.11.3 (26). Sequences were trimmed with Trimmomatic v0.38 (27) for minimum and maximum lengths of 50 and 75 nucleotides, respectively, and a 4-nucleotide sliding window with an average Phred score of 25. Reads passing quality control were aligned to the GRCh38 human genome using HiSAT2 v2.1.0 (28) set to default parameters. FeatureCounts v1.5.0-p2 (29) was employed to count reads aligning to genes, as annotated in Ensembl v93, and counts of those genes uniquely mapped to the human genome were taken forward in the analysis. To investigate samples for contamination with non-human organisms, sequence reads were classified against the NCBI RefSeq database of bacterial, archaeal, viral and eukaryotic microbial genomes, as well as the human genome, using Kraken2 (30).

Further statistical analyses and visualization were performed in R v3.5.0 (31). The EdgeR v3.22.3 (32) package from Bioconductor was used to perform library size normalization (with the trimmed mean of M-values [TMM] method), multi-dimensional scaling, and differential expression analyses (with the glmQFit function). Variation in the data from unknown sources was examined using svaseq (33), and differential expression analysis was performed in a genewise negative binomial generalized linear model in EdgeR that controlled for donor effect (see *Data Availability Statement* for link to BitBucket data repository). Differentially expressed genes were defined by two-fold or greater change, and a Benjamini and Hochberg corrected p-value or false discovery rate (FDR) (34) less than 0.05. Pathway and Gene Ontology enrichment analyses were performed in InnateDB (35). To identify differentially expressed genes associated with estimated viral load, host gene expression was correlated with read counts per million assigned to the ZIKV genome in each sample by Kraken2 (Spearman correlation, p-value less than 0.05). Transcription factor binding site analysis was performed using the findMotifs.pl program in HOMER v4.10 (36), with the human promoter set. A network of molecular interactions between differentially expressed genes, products encoded by those genes and/or their first neighbor interactors was constructed using the Contextual Hub Analysis Tool (CHAT) (37) in Cytoscape v3.8.1 (38), with annotations by Interferome v2.0 (39) and InnateDB (35). This tool identifies “nodes” in the network that are more highly connected to differentially expressed genes than is expected by chance alone (FDR less than 0.05).

## Results

**Figure 1:**
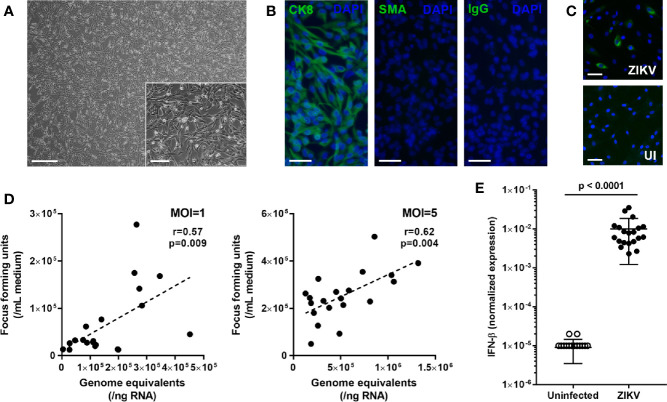
**(A)** Light photomicrograph of human iris pigment epithelial cells immediately prior to infection. Original magnifications: 40x (main image) and 100x (inset image); Scale bars: 500 µm (main image) and 200 µm (inset image). **(B)** Fluorescence photomicrographs of human iris pigment epithelial cells immunolabeled to detect presence of cytokeratin 8 (CK8) and absence of alpha-smooth muscle actin (SMA), with negative control labeled with species-matched immunoglobulin (IgG). Alexa Fluor 488 (green) with DAPI nuclear counterstain (blue). Original magnification: 200x; Scale bar: 100 µm. **(C)** Fluorescence photomicrographs of ZIKV-infected and uninfected human iris pigment epithelial cells immunolabeled to detect flavivirus envelope protein. Alexa Fluor 488 (green) with Hoechst 33342 nuclear counterstain (blue). Original magnification: 100x; Scale bar: 100 µm. **(D)** Correlation plots showing cellular viral load in genome equivalents and infectious foci generated from culture supernatant for human iris pigment epithelial cells infected for 24 hours with ZIKV (multiplicity of infection = 1 and 5). Circles represent individual cell isolates from 20 donors. Line indicates trend. Data were analyzed by Pearson correlation coefficient. **(E)** Graph presenting normalized expression of interferon (IFN)-β transcript, calculated relative to peptidylprolyl isomerase A (PPIA) and ribosomal protein lateral stalk subunit P0 (RPLP0), in human iris pigment epithelial cells infected for 24 hours with ZIKV (multiplicity of infection = 5). Circles represent individual cell isolates from 20 donors. Crossbars indicate mean, and error bars indicate standard deviation. Data were analyzed by Wilcoxon signed-rank test.

Iris pigment epithelial cells were sourced from 12 men and 8 women cadaveric donors, whose ages at death ranged from 44 to 78 years (median = 61.5 years). Time from death to processing of the human eyecups ranged from 7 to 23 hours (median = 12.5 hours), and iris pigment epithelial cells were isolated over a period of 13 to 19 days (median = 16 days). The cells presented as rounded bodies, with some spindle morphology when plated subconfluent ahead of infection (**Figure 1A**). Across all 20 isolates, cytoimmunofluorescence on cells from the same passage as used for subsequent infections indicated homogeneous expression of cytokeratin-8 and complete absence of α-smooth muscle actin, indicating highly pure epithelial cell isolates with no evidence of mesenchymal differentiation (**Figure 1B**).

To investigate the susceptibility of human iris pigment epithelial cells to infection with ZIKV, subconfluent cell monolayers were exposed to the PRVABC59 strain for 24 hours at an MOI of 1. Cytoimmunofluorescent labeling of cell monolayers for the flavivirus ENV identified ZIKV in all 20 isolates following exposure, with viral antigen detected in between 4.4% and 43.2% (median = 14.3%) of cells across the isolates (**Figure 1C**). The RT-qPCR analysis of cellular RNA and focus forming assay of culture supernatant also indicated productive infection of cells at 24 hours, with obvious variation in susceptibility between the 20 isolates, and this result was replicated at an MOI of 5 (**Figure 1D**). For an initial assessment of the type I IFN response of human iris pigment epithelial cell to infection with ZIKV, IFN-β was measured by RT-qPCR in isolates infected at an MOI of 5. This indicated substantial, but variable, increase in cellular IFN-β transcript following infection (**Figure 1E**). These results demonstrated that human iris pigment epithelial cells were moderately susceptible to ZIKV infection, and suggested that this cell population mounted a strong anti-viral response. The results also indicated considerable variation in these parameters across cells isolated from eyes of different individuals.

To explore the molecular response of human iris pigment epithelial cells to infection with ZIKV at a whole transcriptome level, RNA-Seq was performed on total RNA extracted from the 20 primary cell isolates 24 hours following infection with the PRVABC59 strain at an MOI of 5. Across the samples from uninfected isolates, there were 33.6 x 10^6^ mean paired reads, with 33.4 x 10^6^ (99.5%) of reads aligned to the human genome, and 25.0 x 10^6^ (74.5%) mean paired reads assigned to annotated genes. After correction for library size differences, reads aligned to the flavivirus genome in samples infected with ZIKV averaged 9.9% (range = 2.0-24.9%) (**Supplementary Figure 1**). Comparison with a RefSeq database of bacterial, archaeal, viral and eukaryotic genomes indicated negligible contamination with other organisms, including *Mycoplasma* species. Alignment statistics are presented in **Supplementary Table 1**.

**Figure 2:**
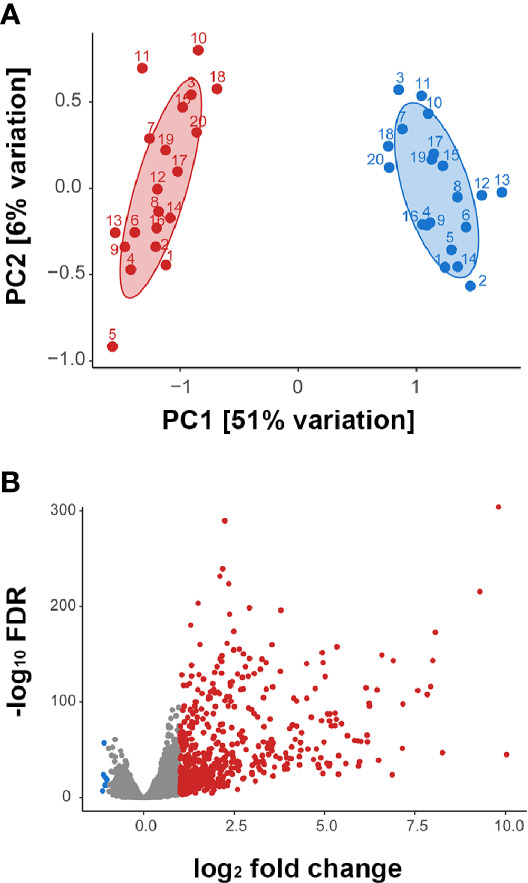
**(A)** Multidimensional scaling plots showing global gene expression in ZIKV-infected (red) and uninfected (blue) human iris pigment epithelial cell isolates. Numbers indicate the 20 individual donors from whose eyes each isolate was generated. PC = principal component. **(B)** Volcano plot displaying differentially expressed genes between ZIKV-infected and uninfected human iris pigment epithelial cells, based on normalized counts per million (false discovery rate < 0.05 and fold change > 2). Red dots indicate significantly upregulated transcripts, and blue dots indicate significantly downregulated transcripts.

**Figure 3:**
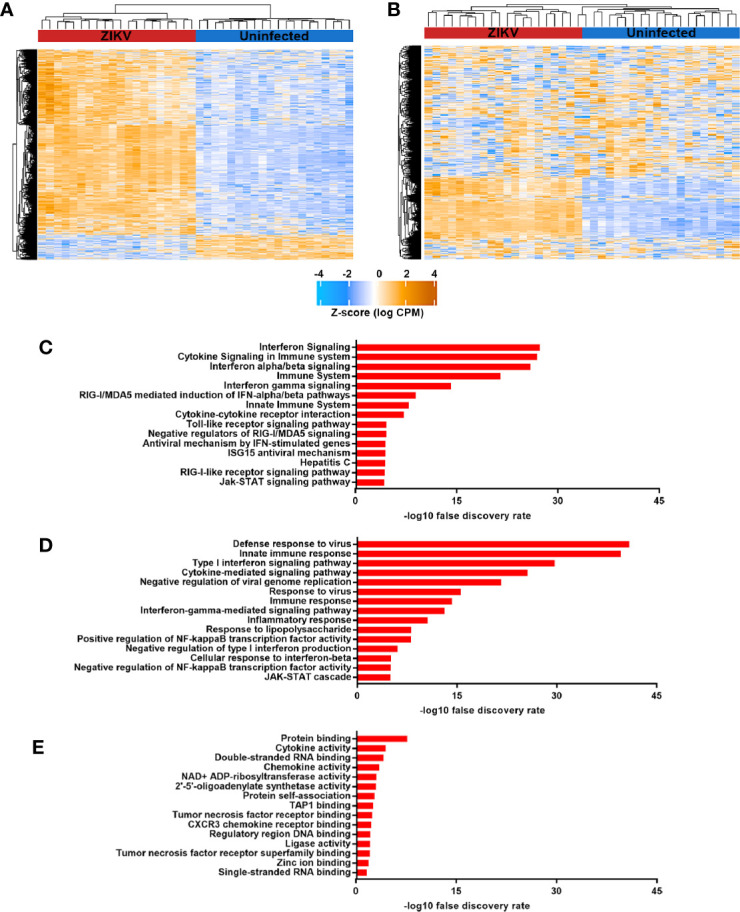
**(A)** and **(B)** Heat maps indicating normalized counts per million from sequencing of total RNA from ZIKV-infected (red) and uninfected (blue) human iris pigment epithelial cells isolated from eyes of 20 individual donors. Z-score is shown for **(A)** all differentially expressed genes and **(B)** the 1,000 most variable genes. Color scale changes from orange to blue, with higher intensity orange representing higher counts for each transcript. **(C–E)** Graphs presenting the 15 most enriched categories –by **(C)** Reactome or KEGG pathways, **(D)** Gene Ontology biological processes and **(E)** Gene Ontology molecular functions– for genes that were upregulated in ZIKV-infected cells, as annotated in InnateDB (35).

Multidimensional scaling of the gene expression data indicated clear separation of the total RNA transcriptome of ZIKV-infected versus non-infected human iris pigment epithelial cell isolates (**Figure 2A**). Data visualization with svaseq (33) indicated that the largest source of variation in the data related to donor (Kruskal Willis, p = 0.005), but this was not explained on the basis of donor age at death or sex, or time from death to cell isolation, and also was not related to batch. Thus, in order to focus on the common cellular response to ZIKV, differential expression analysis was performed controlling for the impact of donor. A total of 7,935 genes (47% of all annotated genes) were differentially expressed between infected and uninfected cells at FDR less than 0.05, including 606 genes that were increased and 7 genes that were decreased at least two-fold in the infected cells (**Figure 2B**, **Supplementary Table 2**). Heat maps showed obvious separation of the set of all differentially expressed genes between ZIKV-infected and uninfected samples (**Figure 3A**), although the 1,000 most variable genes were less discriminating (**Figure 3B**).

Pathway and Gene Ontology biological process analyses of genes significantly upregulated (ie. two-fold or greater change and FDR less than 0.05) in ZIKV-infected iris pigment epithelial cells compared to uninfected cells revealed a strong transcriptional response to infection, involving over 200 different pathways or processes. Molecules involved in immune functions were highly enriched among differentially expressed genes. More specifically, differentially expressed genes were enriched for groupings that included activation of pathogen recognition systems –with retinoic acid-inducible gene I (RIG-I)-like receptor (RLR) and toll-like receptor (TLR) signaling– and anti-viral and inflammatory cytokine responses –with type I and type II IFN signaling (**Figures 3C, D**, **Supplementary Table 3**). Gene ontology molecular function analysis of differentially expressed genes similarly indicated an active response to the virus. Highly enriched functions included broad terms, such as cytokine and chemokine activities, as well as more specific categories, such as single- and double- stranded RNA binding –signifying viral recognition– and 2′,5′-oligoadenylate synthetase (OAS) activity and tumor necrosis factor (TNF) receptor binding –consistent with anti-viral responses (**Figure 3E**, **Supplementary Table 3**). Transcription factor binding site analysis identified 13 regulatory motifs that were enriched in promoter regions of significantly upregulated genes, including multiple IFN-regulating factors (**Table 2**). No binding sites were enriched in genes with significantly downregulated expression.

**Table 2:** Transcription factor binding sites that were enriched in promoters of highly up-regulated genes (target sequences) in comparison with all genes (background sequences) in human iris pigment epithelial cells 24 hours following infection with ZIKV.

Motif (Family)	Consensus sequence	Number of target sequences with motif (%)	Number of background sequences with motif (%)	False discovery rate
ISRE (IRF)	AGTTTCASTTTC	67 (9.6)	427.5 (1.0)	<0.0001
IRF1 (IRF)	GAAAGTGAAAGT	85 (12.2)	864.1 (2.0)	<0.0001
IRF2 (IRF)	GAAASYGAAASY	76 (10.9)	759.3 (1.8)	<0.0001
p65/RELA (RHD)	GGAAATTCCC	29 (4.2)	404.1 (1.0)	<0.0001
IRF4 (IRF)	ACTGAAACCA	71 (10.2)	2057.2 (4.8)	<0.0001
T1ISRE (IRF)	ACTTTCGTTTCT	10 (1.4)	42.6 (0.1)	<0.0001
bZIP-IRF (bZIP, IRF)	NAGTTTCABTHTGACTNW	61 (8.7)	1706.1 (4.0)	<0.0001
p65/RELA (RHD)	WGGGGATTTCCC	95 (13.6)	3490.7 (8.2)	<0.0001
PU.1-IRF (ETS, IRF)	MGGAAGTGAAAC	194 (27.8)	8745 (20.5)	0.0001
PRDM1 (Zf)	ACTTTCACTTTC	78 (11.2)	2838.8 (6.6)	0.0002
Tbx20 (T-box)	GGTGYTGACAGS	28 (4.0)	885 (2.1)	0.0234
Meis1 (Homeobox)	VGCTGWCAVB	167 (23.9)	8238.7 (19.3)	0.0331

BZIP, basic leucine zipper domain; ETS, erythroblast transformation-specific; IRF, interferon regulatory factor; ISRE, interferon-stimulated response element; Meis1, Meis homeobox 1; NF-kB subunit; PRDM1, PR domain-containing protein 1; PU.1, PU box binding-1; RELA, RelA protooncogene, RHD, rel homology domain; T1ISRE, type I interferon-stimulated response element; Tbx20, T-box transcription factor 20; Zf, zinc finger.

**Figure 4:**
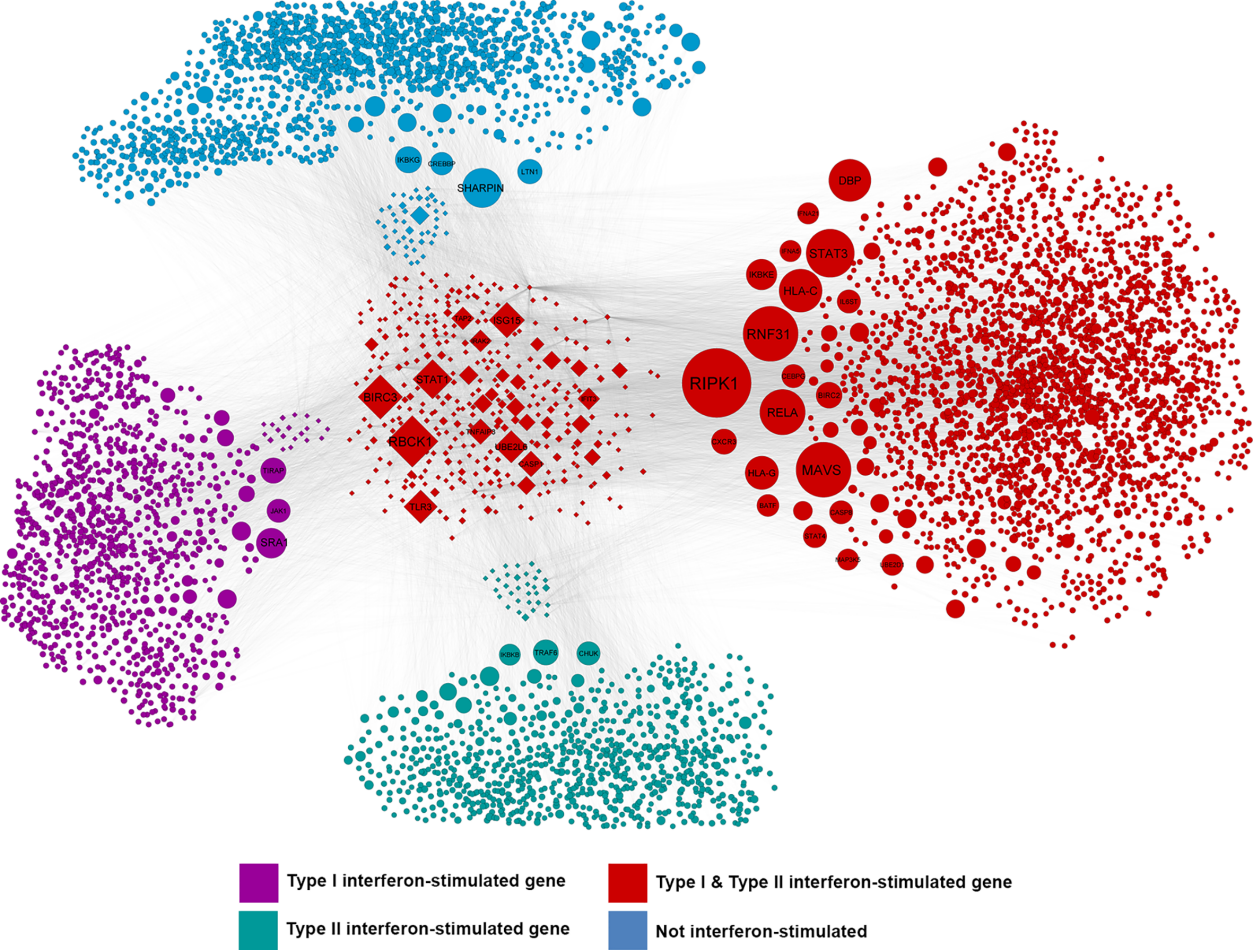
Network of interactions between differentially expressed genes and the first-order interactors of these genes or their encoded products in ZIKV-infected human iris pigment epithelial cell isolates from 20 individual donors. CHAT (37) identified nodes in the network that were connected to differentially expressed genes more than expected by chance. Node size is scaled to -log_10_ of the false discovery rate where larger nodes are those that were identified as being more statistically significant. Purple nodes are type I interferon (IFN)-stimulated genes (ISGs); green nodes are type II IFN ISGs; red nodes are type I and II IFN ISGs; blue nodes are not IFN-responsive, as annotated by Interferome v2.0 (39). Diamond-shaped nodes represent differentially expressed genes (false discovery rate < 0.05 and fold-change > 2). Edges between nodes represent molecular interactions, as annotated by InnateDB (35). Type I and II IFN ISGs were statistically over-represented in this network. Hypergeometric test, p < 0.0001.

A system-level CHAT network (37) was built around genes that were significantly differentially expressed between ZIKV-infected and uninfected human iris pigment epithelial cells, including first-neighbor interactors. This network included 6275 nodes (**Figure 4**, **Supplementary Table 4**). There were 24 contextual hubs that interacted with differentially expressed genes more frequently than predicted by chance alone (**Table 3**). The most highly connected contextual hubs –with greater than 15 contextual neighbors and greater than 200 total neighbors– were members of the Janus kinase (JAK)-signal transducer and activator of transcription (STAT), or nuclear factor κ-light-chain-enhancer of activated B cells (NF-κB) intracellular signaling pathways, indicating their involvement in type I IFN anti-viral responses and inflammation. These six hubs included: inhibitor of NF-κB kinase, regulatory subunit γ (IKBKG); rela protooncogene, NF-κB subunit (RELA); TNF receptor-associated factor 6 (TRAF6); STAT1; STAT3; and ubiquitin-like modifier ISG15 (ISG15). Receptor-interacting serine/threonine kinase 1 (RIPK1) was the most significantly connected contextual hub (p = 5.49 x 10^-5^), with 16 contextual neighbors and 102 total neighbors.

**Table 3:** Contextual hubs (highly connected molecular nodes) that were found to interact with differentially expressed genes more frequently than expected by chance in human iris pigment epithelial cells 24 hours following infection with ZIKV.

Contextual hub	Contextual neighbors	Total neighbors	False discovery rate
RIPK1	16	102	5.49E-05
RNF31	13	83	4.68E-04
MAVS	14	96	4.68E-04
RBCK1	12	75	8.10E-04
STAT3	24	297	0.0013
RELA	25	329	0.0020
BIRC3	12	87	0.0024
HLA-C	11	75	0.0029
DBP	5	11	0.0034
SHARPIN	8	41	0.0052
STAT1	18	207	0.0052
ISG15	18	218	0.0093
HLA-G	5	15	0.0129
TLR3	7	35	0.0129
UBE2L6	8	49	0.0151
IKBKE	11	98	0.0196
SRA1	7	39	0.0219
IKBKG	25	417	0.0377
BIRC2	13	146	0.0400
CASP1	7	44	0.0415
TNFAIP3	10	92	0.0428
TIRAP	7	45	0.0436
CXCR3	3	5	0.0446
TRAF6	21	330	0.0446

BIRC3, baculoviral IAP repeat-containing protein; CASP1, caspase 1, apoptosis-related cysteine protease; CXCR3, chemokine, CXC motif, receptor 3; DBP , d-box-binding PAR bZIP transcription factor; HLA , major histocompatibility complex, class I; IKBK , inhibitor of nuclear factor kappa-B kinase; ISG15, ubiquitin-like modifier ISG15 (a.k.a. interferon-induced protein 15); MAVS, mitochondrial antiviral signaling protein; RBCK1, RANBP-type and C3HC4-type zinc finger-containing 1; RELA, rela protooncogene, NFKB subunit; RIPK1, receptor-interacting serine/threonine kinase 1; RNF31, ring finger protein 31; SHARPIN, shank-associated RH domain interactor; SRA1, steroid receptor RNA activator 1; STAT, signal transducer and activator of transcription; TIRAP, TIR domain-containing adaptor protein; TLR3, toll-like receptor 3; TNFAIP3, tumor necrosis factor-alpha-induced protein 3; TRAF6, TNF receptor-associated factor 6; UBE2L6, ubiquitin-conjugating enzyme E2L 6.

**Figure 5:**
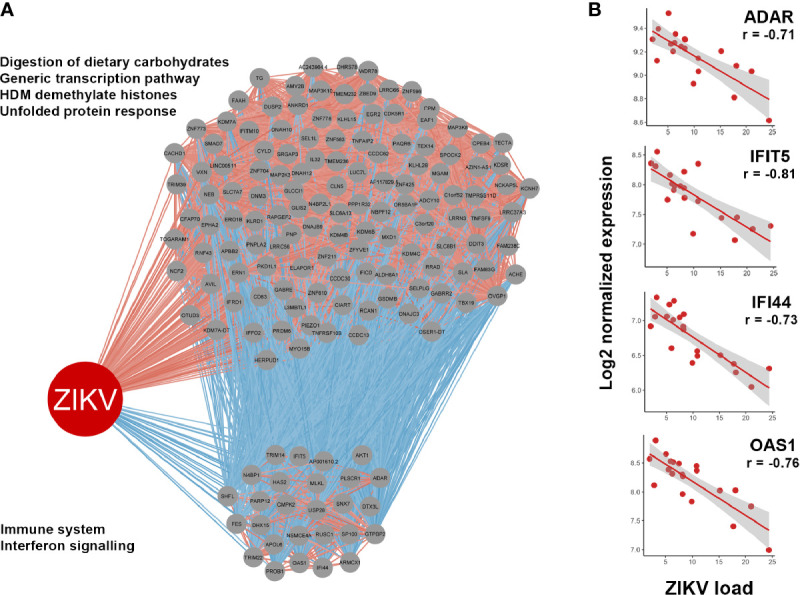
**(A)** Correlations between genes upregulated in ZIKV-infected human iris pigment epithelial cells and read counts per million assigned to the ZIKV genome (ZIKV load). Spearman correlation, p < 0.05. Gray circles indicate upregulated genes. Red lines indicate positive correlations, and blue lines indicate negative correlations. Enriched Reactome pathways achieving false discovery rate < 0.05 are listed left of the relevant grouping, with annotations by InnateDB (35). **(B)** Examples of negative correlations between interferon (IFN)-stimulated genes and ZIKV load. Red dots indicate 20 individual donors. Line indicates trend. ADAR, adenosine deaminase, RNA-specific; IFI44, IFN-induced protein 44; IFIT5, IFN-induced protein with tetratricopeptide repeats 5; OAS1, 2’-5’-oligoadenylate synthetase 1.

Associations between gene expression by the host cell and intracellular ZIKV load were identified by correlating upregulated genes with normalized read counts assigned to the viral genome in the infected iris pigment epithelial cell isolates across the 20 human donors. This analysis identified 146 upregulated genes that were significantly correlated with read counts assigned to ZIKV, including 122 that were positively correlated and 24 that were negatively correlated (**Supplementary Table 5**). Reactome pathway enrichment analysis of these genes indicated ‘IFN signaling’ and ‘immune system’ were negatively correlated with ZIKV read counts, while cell cycle-related pathways were positively correlated (**Figure 5A**). Examples of upregulated IFN-stimulated genes that were negatively correlated with ZIKV load included: adenosine deaminase, RNA-specific (ADAR); IFN-induced protein 44 (ITI44); IFN-induced protein with tetratricopeptide repeats 5 (IFIT5); and OAS1 (**Figure 5B**).

## Discussion

Zika virus disease is often asymptomatic or manifest as a mild and self-limited systemic illness in adult patients. However, serious inflammatory complications may occur, and these include anterior uveitis, which has been linked with presence of ZIKV in the anterior eye (8, 9). Our work represents the first research to examine mechanisms of Zika-associated anterior uveitis, focusing on the interactions between the virus and iris pigment epithelial cells, and using the PRVABC59 strain isolated during the American epidemic and primary cells isolated within 24 hours of death from human cadaveric donors. Human iris pigment epithelial cell isolates generated from 20 donors were permissive to ZIKV infection, and supported replication and release of the virus. RNA-Seq indicated a vigorous molecular response of the host cell to ZIKV. Almost 50% of annotated genes were differentially expressed between infected and uninfected cells, and 99% of the 613 genes that changed at least two-fold were up-regulated. Pathway, Gene Ontology and network analyses indicated strong activation of viral recognition and defense, as well as biosynthesis mechanisms consistent with production of virus. Although all iris pigment epithelial cell isolates mounted a type I IFN response, intracellular virus load correlated inversely to overall IFN signaling across the donors.

When uveitis occurs in the context of a systemic infectious disease, the pathology usually represents a combination of tissue damage induced by the microbe and reactive inflammation by the host. Although some forms of infection-associated uveitis are believed to be primarily immune-mediated (40), recent widespread molecular testing of ocular fluids has demonstrated that a broad range of both dangerous and innocuous viruses access the eyes in immunocompetent persons to cause uveitis –for example, cytomegalovirus (CMV), rubella virus and Ebola virus (EBOV) (41–43). ZIKV also has been recovered from the aqueous humor in anterior uveitis (**Table 1**). Studies in mice suggest that ZIKV accesses the eye from the blood stream in myeloid cells that become hypermotile (44, 45), as is also the case for more common ocular pathogens such as *Toxoplasma gondii* (46). Various ocular cell populations are preferred hosts for different organisms, including retinal pigment epithelium for EBOV and *Mycobacterium tuberculosis* (47, 48), and retinal Müller glial cells for CMV and *T. gondii* (49, 50). Given that ZIKV causes serious retinal pathology *in utero*, previous research in the field has focused on cells of the posterior eye, including Müller cells, retinal pigment epithelial cells and retinal vascular endothelial cells (17, 19, 51). Iris pigment epithelial cells are key players in inflammation of the anterior segment of the eye, which defines anterior uveitis. They have a dual role, as key regulators of ocular immune privilege (20), and as the source of an array of inflammatory cytokines (52). Our data indicate that ZIKV is capable of productively infecting human iris pigment epithelial cells.

While all 20 primary human iris pigment epithelial cell isolates were susceptible to infection with ZIKV, infectivity varied across donors, as demonstrated by RT-qPCR measurement of intracellular viral load and a focus forming assay of supernatant collected from cultures of infected cells. Variation in response to infection, as well as at baseline, is likely to be caused by multiple technical and biological factors. A consistent method of cell isolation and culture was used, but donor eyes were necessarily sourced on different dates. There was also unavoidable variation in time from death to cell isolation and time in culture, although these intervals were minimized to avoid mesenchymal transition of cells. Biological variation relates to individual genetics and epigenetics, as well as health and disease status. In work with other primary human ocular cells –including retinal and choroidal endothelial cells, Müller cells and retinal pigment epithelial cells– we (53–55) and other groups (56–59) have demonstrated variation in gene expression by human donor. This highlights the importance of not relying on results from individual cell isolates in drawing conclusions about pathobiological mechanisms. A major strength of our study is the large number of human donors, affording a comprehensive overview of the gene expression of the human iris pigment epithelium by RNA-Seq, while also identifying inter-individual variations.

Intracellular viral recognition is coordinated by two pattern recognition receptor families: TLRs and RLRs (60). Pathway and Gene Ontology analyses indicated high activation of RIG-I and melanoma differentiation-associated protein 5 (MDA5) signaling; TLR3, which recognizes double-stranded RNA specifically, was identified as a contextual hub by CHAT network analysis. Viral defense relies on IFN responses. IFN-β –the prototype type I IFN– was the most highly differentially expressed transcript in infected human iris pigment epithelial cells, with an approximately 900-fold upregulation across the 20 isolates. Enrichment analyses identified activation of type II IFN signaling, as well as type I. Type III IFNs –including IFN-λ1, IFN-λ2 and IFN-λ3– also were substantially increased following infection. The IFNλ receptor is a heterodimer of IL10RB, which is ubiquitously expressed, and IFNLR1, which has considerably restricted expression, but was identified in this cell population by RNA-Seq. The type III IFN system protects barriers (61), including the female reproductive tract in Zika (62). Recent work has identified Type III IFN signaling on the ocular surface, with corneal epithelium also expressing both subunits of IFNλR1 (63). Expression of type III IFNs and IFNR1λ is consistent with the role of iris pigment epithelial cells in maintaining ocular immune privilege. The transcription factor analysis also indicated activity in pathways that would increase IFN-stimulated genes. Interestingly, key players in noncanonical NF-κB signaling were upregulated, suggesting the possibility of negatively regulating induction of type I IFNs (64). NF-κB signaling also would be expected to promote inflammatory responses and trigger a breach in immune privilege, consistent with the development of anterior uveitis.

While anti-viral IFN responses were highly upregulated in ZIKV-infected iris pigment epithelial cells, the intracellular viral load correlated inversely with IFN signaling. Consistently, multiple ISGs were upregulated at a relatively low level in heavily infected isolates. This result suggests ZIKV directly interferes with IFN signaling in epithelial cells. The ZIKV polypeptide yields 7 nonstructural (NS) proteins in addition to 3 structural proteins (65). ZIKV NS5 in particular has multiple actions that target IFN signaling. On binding to RIG-I, NS5 represses the ubiquitination that promotes RIG-I-mediated signaling (66). NS5 binds TANK-binding kinase 1 (TBK1), interfering with its ability to phosphorylate and activate IRF3 ahead of nuclear translocation (67); NS5 also binds IRF3 to limit its activity (68). Together with IRF9, STAT1 and STAT2 form the IFN-stimulated gene actor-3 complex (ISGF-3), but NS5 targets STAT2 for proteosomal degradation, by either binding it directly or binding the E3 ubiquitin ligase, seven in absentia homolog 2 (SIAH2), which subsequently binds STAT2 (69, 70). Other nonstructural proteins also may impact IFN production and/or induction of ISGs (68, 71). While NS5 suppresses type I and III IFN signaling, it enhances type II IFN signaling by destabilizing STAT2 and limiting formation of ISGF-3, and promoting formation of STAT1-STAT1 homodimers that bind γ-activated sequence (GAS) elements (72). Thus, increased viral replication could promote anterior uveitis by increasing type II IFN signaling. NS5 also facilitates the assembly of the NLRP3 (NLR family, pyrin domain-containing 3) inflammasome (73).

The “central controller” of cell fate –RIPK1 (74)– was the most significant contextual hub in the molecular network generated from the list of 613 differentially expressed genes in iris pigment epithelial cells following ZIKV infection. While not differentially expressed per our definition of two-fold change and FDR less than 0.05 (**Supplementary Table 2**), RIPK1 increased highly significantly (FDR = 8 x 10^-30^) by 1.5-fold post-infection. Originally described as an activator of necroptosis, involvement of RIPK1 in different pathological processes is now known to be multifaceted. The protein has a C-terminal death domain, an N-terminal serine/threonine kinase domain, and an intermediate domain that participates in NF-κB activation (74). In addition to necroptosis, RIPK1 may trigger apoptosis and promote inflammation, and conversely, it may act as a regulator of each of these processes (75–77). The ultimate impact of RIPK1 signaling in ZIKV-infected human iris pigment epithelial cells is thus difficult to predict. Recent work in a mouse central nervous system model of ZIKV infection indicated RIPK1 signaling directed a “metabolic reprogramming” in infected neurons characterized by inhibition of succinate dehydrogenase, which suppressed viral replication (78), suggesting RIPK1-controlled activities might have anti-viral effects.

To interrogate the mechanisms of Zika-associated anterior uveitis as it occurs in the patient, we selected an *in vitro* system with iris pigment epithelial cells isolated from human cadaver eyes. Iris biopsy is not performed in the clinical setting, and thus it is not feasible to conduct such research using tissue from patients diagnosed with this condition. Removing the cells from the local microenvironment and the culture procedure is expected to induce some change in cell phenotype. We used early passage cell isolates, all of which were checked for presence of epithelial and absence of mesenchymal markers. The most common *in vivo* models are induced by systemic inoculation of virus into mice, including animals with deficient type I IFN signaling (12–15). A non-human primate model in the Rhesus macaque has recently been described (79). However, these studies have not involved adult animals. Indeed, in descriptions of several mouse models, Zhao and colleagues (13) commented that wild-type mice infected intraperitoneally with ZIKV lost susceptibility to ocular pathology from one week of age, and A129 mice –with null mutations in the IFN-α/β receptor– showed no ocular disease after 10 weeks. Past works also have focused largely on posterior eye pathology. An interesting new experimental model for investigating anterior uveitis involves injecting ZIKV into the aqueous humor of 1-month-old mice; the animals develop raised intraocular pressure, and pattern recognition receptors and inflammatory chemokines are induced in anterior segment tissues (80).

Zika-associated anterior uveitis occurs when ZIKV accesses the eye during a systemic infection. However, the eye is able to clear the virus, and the inflammation resolves either spontaneously or with the short-term use of corticosteroid eye drops. Our work represents the first observations around basic mechanisms of this emerging form of uveitis in a human-based experimental system. Our results suggest that the iris pigment epithelium is susceptible to ZIKV infection, but also mounts a rigorous pan-IFN response, which when combined with other elements of ocular immune privilege, may limit ocular pathology in the majority of affected individuals. Those persons in whom the virus replicates robustly within the iris pigment epithelium, may generate a stronger inflammatory response and thus be at higher risk of developing anterior uveitis.

## Data Availability Statement

The datasets presented in this study can be found in online repositories. Sequence data are available in the Gene Expression Omnibus (GEO) of the National Centre for Biotechnology Information (NCBI) under the accession number GSE131605. Read counts and code are available in a BitBucket data repository: https://bitbucket.org/lynnlab/ZIKV.

## Ethics Statement

Use of human cadaver donor eyes from the Eye Bank of South Australia (Adelaide, Australia) for this work was approved by the Southern Adelaide Clinical Human Research Ethics Committee (protocol number: 175.13). Retrospective collection of the clinical data of individuals diagnosed with ZIKV-associated anterior uveitis at the Ophthalmology Clinic of Ribeirao Preto General Hospital, Ribeirao Preto, Sao Paulo, Brazil, between January 2016 and June 2016, was approved by the Hospital Ethics Committee of Human Research (protocol number: 85195318.0.0000.5540).

## Author Contributions 

JS conceived the study. FR, JC, JF, MM, BA, DL, and JS designed the study. JF and MS collected and synthesized the clinical data. JC, YM, LA, GO, GG, AD, BA and JS performed the laboratory experiments and/or analyzed laboratory data (including contributions to methodology). FR and DL analyzed and interpreted the RNA-Seq data. JS wrote the article including review of the relevant literature. FR and LA contributed substantially to preparation of the figures and/or tables in the article. FR, JC, JF, YM, LA, MS, GO, GG, AD, MM, BA and DL critically reviewed the content of the article. All authors contributed to the article and approved the submitted version.

## Funding

This work was supported by grants from Foundation for Support of Teaching, Research & Assistance of the Clinical Hospital, Faculty of Medicine of Ribeirão Preto - University of São Paulo (FAEPA: 1973/2019 to JF); Avant Mutual Group (Doctor in Training Research Scholarship to GO); EMBL Australia (Group Leader Award to DL); Rebecca L. Cooper Medical Research Foundation (Research Grant to JS); National Health & Medical Research Council (GNT1139857 to JS); and Australian Research Council (FT130101648 to JS).

## Conflict of Interest

The authors declare that the research was conducted in the absence of any commercial or financial relationships that could be construed as a potential conflict of interest.

## Supplementary Material

Supplementary Figure 1 Bar plot showing composition of ZIKV-infected and uninfected human iris pigment epithelial cell samples, as classified by Kranken2 (30) against the GRCh38 human genome and the NCBI RefSeq database of bacterial, archaeal, viral and eukaryotic microbial genomes.

Supplementary Table 1 Alignment Statistics.

Supplementary Table 2 Differential Expression Analysis.

Supplementary Table 3 Enrichment Analysis.

Supplementary Table 4 Network Analysis.

Supplementary Table 5 Correlation Analysis.
