# Supplementary figures and images for: Zika Virus Infection of Human Iris Pigment Epithelial Cells
Source: Front Immunol. 2021 Apr 22;12:644153. doi: 10.3389/fimmu.2021.644153 (PMC8100333; doi:10.3389/fimmu.2021.644153)

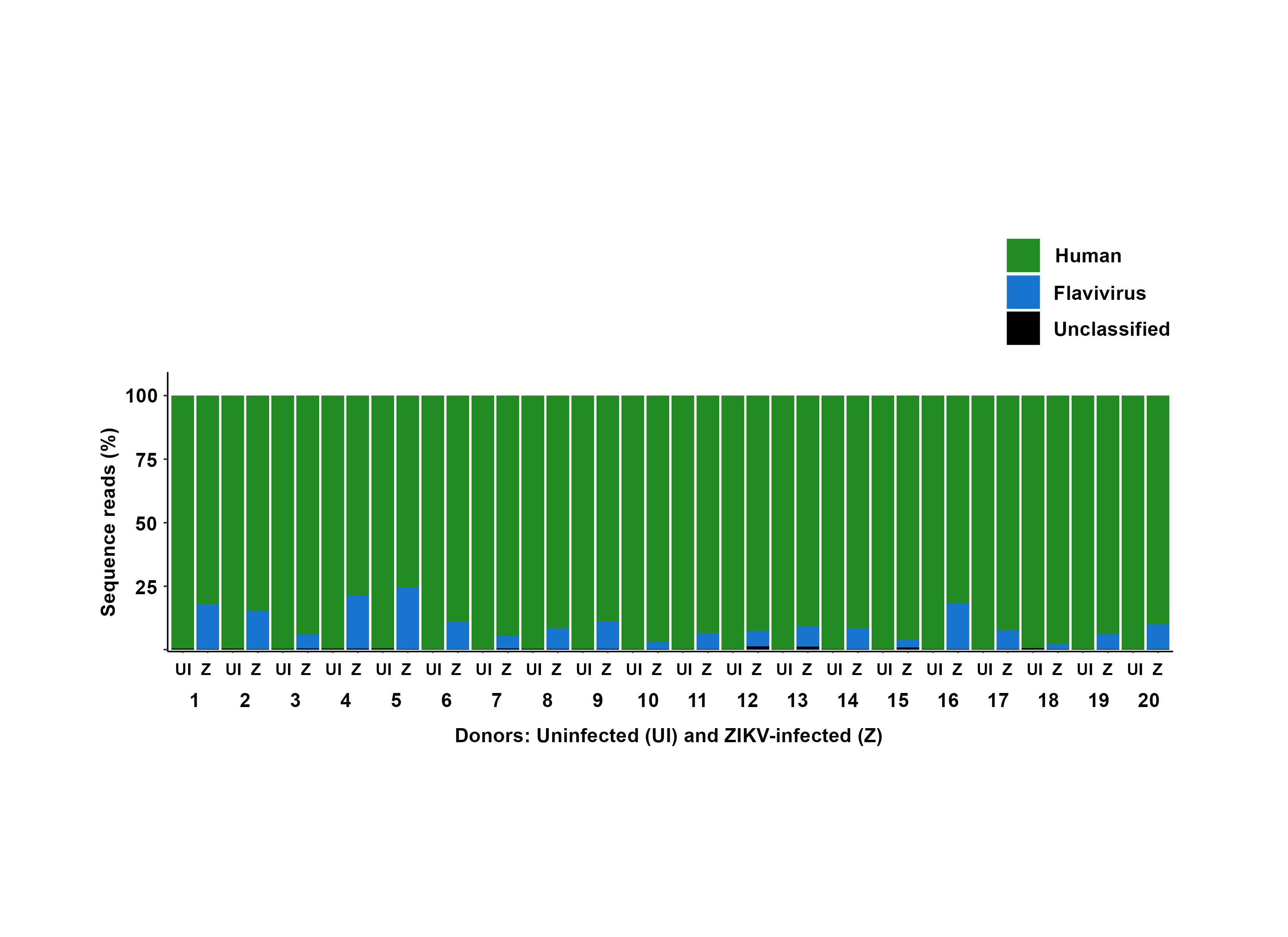

Supplement: Supplementary Figure 1 — Bar plot showing composition of ZIKV-infected and uninfected human iris pigment epithelial cell samples, as classified by Kranken2 (30) against the GRCh38 human genome and the NCBI RefSeq database of bacterial, archaeal, viral and eukaryotic microbial genomes. [file Image_1.tif]
